# Direct Spectrophotometric Method for Determination of Cadmium (II) Ions Using Bis-Thiosemicarbazone

**DOI:** 10.1155/ianc/3347969

**Published:** 2025-05-05

**Authors:** Sulafa Nassar, Gharam I. Mohammed, Thoraya A. Farghaly

**Affiliations:** ^1^Chemistry Department, Faculty of Science, Umm Al-Qura University, Makkah, Saudi Arabia; ^2^Department of Chemistry, Faculty of Science, Cairo University, Giza, Egypt

**Keywords:** *bis*-thiosemicarbazone ligand, Cd (II) ions (II) complex, UV-Vis spectrum

## Abstract

A novel and simple study outlines the advancement of a straightforward and precise spectrophotometric technique for the determination of Cd (II) ions. This method offers a notable benefit as it is a straightforward procedure that does not require additional purification or concentration of the solvent. The concentration of Cd (II) ions was determined in the presence of *bis*(indoline-2, 3-dione) thiosemicarbazone (L) at a pH of 12 using Briton–Robinson Buffer. The concentration range for Cd (II) ions in the method follows Beer's law and is between (1.8–17.8) × 10^−5^ mol L^−1^. The limit of detection is 0.245 μg mL^−1^(2.2 μmol L^−1^) and the limit of quantification is 0.817 μg mL^−1^ (7.3 μmol L^−1^). The molar ratio between L and Cd (II) ions was 1:2, ensuring the development of a metal complex. The applied method offers numerous benefits, including its simplicity, affordability, convenience of use, quick detection, minimal use of ligands, and high sensitivity. The sensitivity of the analytical approach was verified by carefully selecting appropriate experimental conditions. Additional insights into the composition and arrangement of the complex produced in a solution containing Cd (II) ions and the ligand (L) have been obtained by isolating and studying the solid complex L-Cd. The solid complex, L-Cd, was determined using analytical methods including elemental analysis, UV-Vis spectra, spectral mass, and thermal analysis.

## 1. Introduction

The majority of known heavy metals are toxic and dangerous, particularly cadmium (Cd (II) ions) [1–3]. It poses a threat to both humans as well as animals. It occurs undoubtedly in the environment as a contaminant resulting from agricultural and industrial activities. The most common sources of cadmium contamination in the environment are mining, dyes, metals, the manufacture of nickel and cadmium batteries, and plastic stabilizers [4–6]. Smoking and being subjected to high levels of Cd (II) ions can also result in poisoning and a variety of deadly cancers [7, 8]. Cadmium, on the other hand, causes DNA damage by increasing the generation of ROS, or reactive oxygen species [9, 10]. Cd (II) ions also modify gene expression and inhibit enzyme activity in antioxidant defense systems [11]. Heavy metals can accumulate in hair, soft tissues (such as kidneys, liver, brain, or lungs), as well as bones [12, 13]. Measuring cadmium levels in water, agricultural, and biological material is crucial for the safety of humans and the environment. Several techniques for determination of the metals were spread and expanded quickly including AAS [14], spectrophotometry [15, 16], flow injection [17], chemiluminescence [18], polarography [19], ICP-MS [20], and ICP-AES [21]. The most common technique is direct visible absorption spectrophotometry, which is a widely utilized technique owing to its nondestructive characteristics, rapid measurement capabilities, ease of use, minimal data processing requirements, and cost-effectiveness. Nevertheless, it possesses vulnerabilities including stray light, light scattering, interference from various absorbing species, and geometric factors [22]. On the other side, thiosemicarbazone derivatives have received the attention of many scientists due to their ability to coordinate with metal ions [23, 24]. This is because it is distinguished by the presence of oxygen, nitrogen, and sulfur atoms that donate electrons [25]. Many references have proven the ability of thiosemicarbazone derivatives to coordinate with metal ions and produce complexes that have many applications in various industrial, agricultural, and medical fields [26–28]. Therefore, thiosemicarbazones have vital role in contribution to detect several types of metal ions in their aqueous solutions through coordination [29]. Moreover, the efficiency of *bis*-thiosemicarbazone derivatives through detection and determination of the metal ions will be more efficient due to the presence of two chelating centers to coordinate with the metals at the same time. From all the above findings, the present work based on detection and determination the cadmium ions in solution through the quick, straightforward, and inexpensive colorimetric method via the formation of complex with *bis*-thiosemicarbazone derivative. Also, the stoichiometric of L-Cd complex was detected using molar ration, NMR, and IR methods.

## 2. Experimental

### 2.1. Chemicals and Reagents

All previously used laboratory equipment was thoroughly rinsed with a 10% HNO_3_ solution (Germany) before each use. A concentrated solution of cadmium ions (8.9 × 10^−3^ mol L^−1^) was made by combining 2.03 g of cadmium chloride dihydrate CdCl_2_.2H_2_O in 100 mL of ultra-pure distilled water according to the exact weight. Then, using the dilution procedure, additional Cd (II) ion solution dilutions ranging from 1.8 to 17.8 × 10^−5^ mol L^−1^ were created. Additionally, 0.1 g of *bis*(indoline-2, 3-dione) thiosemicarbazone (L) was dissolved in 100 mL of 1, 4-dioxane (MFF, Hayman, England) to create a ligand stock solution. A mixture of 0.04 mol L^−1^ CH_3_COOH, 0.04 mol L^−1^ H_3_PO_4_, and 0.04 mol L^−1^ H_3_BO_3_ was used to make a quantity of Britton–Robinson (B-R) buffer (pH 3–12). The pH was subsequently modified using 0.2 mol L^−1^ of NaOH [30].

### 2.2. Instruments and Equipment's

The absorption spectra were recorded using spectrophotometer (a Shimadzu UV-1800 model UV-Vis). The quartz cells used in the experiment had a one-cm bath length. A Mettler Toledo MP220 pH meter was employed to determine the solutions' pH.

### 2.3. Recommended Procedures

Concentrations of Cd (II) ion solutions ranging from 1.8 to 17.8 × 10^−5^ mol L^−1^ were prepared. Additionally, 0.5 mL of a ligand (L) solution with a concentration of 2.1 × 10^−3^ mol L^−1^ was added to 10 mL volumetric flasks. The resultant solutions were made alkaline with a pH of 12 by adding B-R buffer, and then the volume was completed to 10 mL using an aqueous solution. Every solution's absorbance was measured in relation to a blank solution (Ligand) constructed in the same manner.

### 2.4. Synthesis of Ligand (L)

**Figure 1:**
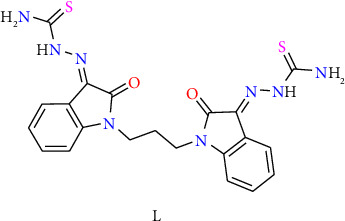
Structure of the *bis*-thiosemicarbazone ligand L.

The *bis*-thiosemicarbazone ligand named *bis*(indoline-2, 3-dione) thiosemicarbazone L (Figure 1) has been prepared with identical method previously reported in our laboratory [31].

### 2.5. Synthesis of Cd (II) Ions Complex (L-Cd)

Cd (II) ions complex of the *bis*(indoline-2, 3-dione) thiosemicarbazone L was prepared through the addition of a hot ethanolic solution of 0.456 g of CdCl_2_.2H_2_O (0.002 mol) in 15 mL ethanol to a clear dissolved solution of 0.48 g of *bis*(indoline-2, 3-dione) thiosemicarbazone L (0.001 mol) in 30 mL dioxane, the whale solution was reflux for 2h. Et_3_N (1 mL) was added after 2 hours of reflux then the mixture was left to reflux another 2 h. The pale-yellow solid formed was gathered with filtration. The formed Cd (II) ions-complex was rinsed several times utilizing ether then it was dried in vacuum over CaCl_2_-anhydrous. The yield of the Cd (II) ions-complex was 71% with pale yellow color.

[LCd_2_Cl_4_(H_2_O)_2_] Molecular formula is C_21_H_24_Cd_2_Cl_4_N_8_O_4_S_2_ (Mol. Wt.: 883.23): C, 28.56; H, 2.74; Cd (II) ions, 25.45; N, 12.69. Found: C, 28.37; H, 2.51; Cd (II) ions, 25.29; N, 12.43%. IR (cm^−1^, KBr disc): 3524 (br. NH_2_, NH), 1748 (*υ* (C=O)), 1620 (*υ*(C=N)), 1338 (*υ* (C=S)), 727 (*υ* (M−O)), 457 (*υ* (M−N)).

## 3. Results and Discussion

### 3.1. UV-Vis Spectrum of Cd (II) Ions Complex in Solution

**Figure 2:**
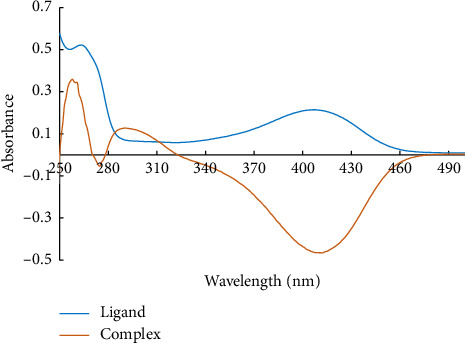
Absorption spectra of 4.2 × 10^−5^ mol L^−1^ of L and 1.8 × 10^−5^ mol L^−1^ of Cd (II) ions complex solution at pH = 12.

UV-Vis spectra of the ligand and the Cd (II) ion complex, L-Cd, were recorded in deionized water (D.W.). Two bands that appeared at 265 nm (as a shoulder) and 410 nm in the UV-visible spectrum of ligand are assigned to π⟶⁣π^∗^. During the addition of the Cd (II) ions solution, the ligand's deep orange color transformed into an orange-yellow, resulting in the appearance of a peak absorption at 290 nm (blue shift) when compared to the ligand and showing clear evidence of C = N coordination to cadmium, as depicted in Figure 2. Based on these findings, a wavelength of 290 nm was selected for the detection of Cd (II) ions.

### 3.2. Analytical Parameters Optimization

pH is a crucial analytical parameter for synthesizing metal complexes and needs to be studied. The effect of pH in the 2.0–12.0 range was examined using B-R buffer. A steady and maximal absorbance signal was only achieved at pH 12. This result suggests that the formation of the cadmium (II) ion complex is significantly influenced by the basicity of the solution. Consequently, the ideal conditions were determined to be a pH of 12.

At pH 12, the Cd (II) ion complex's absorbance peaked. The interaction between the chelating reagent (L) and the analyte (Cd (II) ions) can only take place if binding sites for the ligand are present, which is why this behavior occurs. Also, because there are more OH^−^ ions and fewer H^+^ ions at higher pH, the chelating agent's phenolic OH groups are more easily deprotonated. The ligand's binding to the Cd (II) ions is made easier by this.

**Figure 3:**
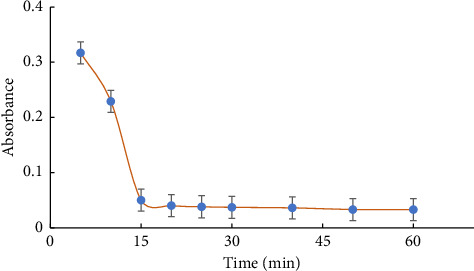
Correlation between absorbance of the L-Cd complex and time. Conditions: 8.9 × 10^−5^ mol L^−1^ Cd (II) ions solution in existence of L with the concentration 1.6 × 10^−4^ mol L^−1^ at 290 nm with pH 12.

The stability of the formed compound was investigated under optimal analytical conditions. An investigation was conducted to examine the influence of time on the liberation of the complex, with a duration ranging from 5 to 60 min. Upon formation of the complex, the absorbance exhibited a gradual increase over time, peaking after 5 min. Subsequently, the absorbance of the complex started to decrease, as depicted in Figure 3. Consequently, under ideal conditions, the reactants were mixed, and the highest level of absorbance was attained after 5 min of stability for the creation of the complex.

**Figure 4:**
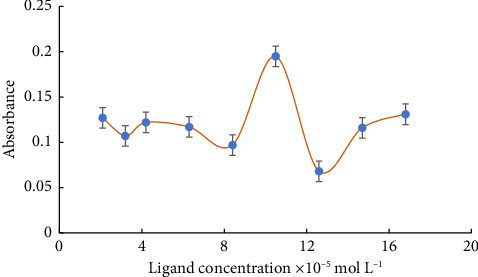
The effect of the concentration of the ligand on the absorbance of 8.9 × 10^−5^ (mol L^−1^) Cd (II) ions at 290 nm and pH = 12.

Among the parameters that have also been evaluated and their effect on analytical response has been investigated is ligand concentration. So, at pH 12, a set of concentrations of ligand (L) solutions in the regain between 2.1 × 10^−5^ and 16.8 × 10^−5^ mol L^−1^ were included in the solution of the metal ion. The ideal concentration of the ligand was found to be 10.5 × 10^−5^ mol L^−1^ based on the findings derived from Figure 4.

### 3.3. Analytical Performance

**Figure 5:**
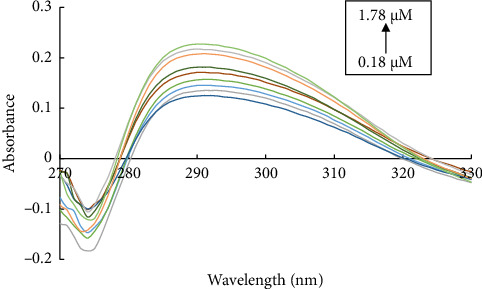
Absorption spectra of the formed L-Cd and various concentrations of Cd (II) ions ((1.8–17.8) × 10^−5^ mol L^−1^) upon the existence of ligand with 10.5 × 10^−5^ (mol L^−1^) concentration at pH 12.

The analytical efficacy of the proposed approach was tested under previously established ideal conditions, which are dependent on the creation of a yellow-orange complex by the interaction of L with Cd (II) ions in an aqueous solution.  Figure 5 depicts the absorption spectra of the Cd (II) ion complex under ideal conditions and varying Cd (II) ion concentrations (ranging from 1.8 to 17.8 × 10^−5^ mol L^−1^). A graph showing the relationship between Cd (II) ion concentration and the complex's absorbance at 290 nm proved that the Beer–Lambert equation was followed. Recovery experiments were used to evaluate the method's accuracy and precision.

**Figure 6:**
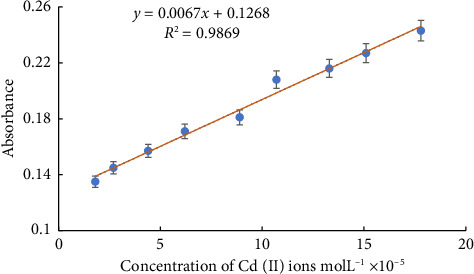
Calibration curve of L-Cd complex with optimal conditions: (Cd [II] ions) = (1.8–17.8 × 10^−5^) mol L^−1^ in 10.5 × 10^−5^ mol L^−1^ ligand and pH = 12, at λ_max_   = 290 nm.

The absorbance of the Cd (II) ion complex at 290 nm was plotted toward the Cd (II) ion concentration. The correlation coefficient of such linear relationship was found to be 0.9869 for this plot. The concentration range for this analysis was (1.8–17.8) × 10^−5^ mol L^−1^, as illustrated in Figure 6.

The linear plot can be expressed as the following equation:

(1) $$\begin{array}{c} A=0.0067C\left(\text{mol }L^{-1}\right)+0.1258\left(r^{2}=0.9869\right). \end{array}$$

The molar absorptivity of the Cd (II) ions complex was found to be 6.7 × 10^2^ L mol^−1^ cm^−1^ as calculated from Beer's–Lambert plot.

The analytical approach's sensitivity can be assessed using the parameters of the limit of quantification (LOQ) and limit of detection (LOD). In order to calculate LOD and LOQ [32], the following formulas were applied, with the curve of calibration serving as the basis:

(2) $$\begin{array}{c} \text{LOD}=\frac{3\sigma }{S}, \end{array}$$

(3) $$\begin{array}{c} \text{LOQ}=\frac{10\sigma }{S}. \end{array}$$

Here, *σ* and S are defined as the response's standard deviation and the calibration curve's slope, respectively.  Table 1 shows that the LOD for the Cd (II) ions complex formation were 0.245 μg mL^−1^ (2.2 μmol L^−1^) and LOQ were 0.817 μg mL^−1^ (7.3 μmol L^−1^). Based on the evaluation of the analytical technique using the Relative Standard Deviation (RSD), it was determined that the precision was 5.1% (*n* = 5).

### 3.4. Stoichiometry of Cd (II) Ions Complex

**Figure 7:**
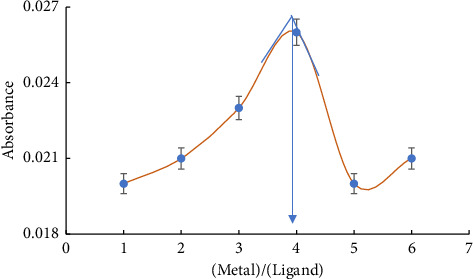
Plot the molar ratio for the liberated L-Cd complex at 290 nm and pH 12.

The stoichiometry of the generated cadmium complex was assessed using the molar ratio technique. The results confirmed that the stoichiometry of the complex is 2 to 1, with cadmium to ligand. It is apparent that 2.0 mol of Cd (II) ions are chelated with 1.0 mol of ligand.  Figure 7 depicts a graphical representation of the outcomes.

**Figure 8:**
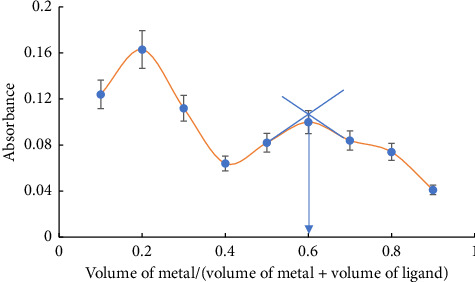
Job's method for continuous variation of the liberated L-Cd complex; 2.1 × 10^−3^ (mol L^−1^) of both Cd (II) ions and L solutions and *λ* of 290 nm.

Using Job's method for continuous variation, the molar ratio was determined, confirming that the L-Cd complex was produced in a molar ratio of 1 (L):2 (Cd (II)). The molar ratio method also came to the same conclusion (Figure 8).

At pH 12, the molar ratio technique successfully determined the stability constant of the colored L-Cd (II) ion complex [33]. Near the equivalency point on the molar ratio plot, the extrapolated absorbance value (A expt) represents the total absorbance of the complex L-Cd.  Figure 7 shows that the complex has a stoichiometry of 2:1 (Cd (II): L), as determined by the equation that follows:

(4) $$\begin{array}{c} M^{n+}+X^{-}{↔\left[\text{MX}\right]}^{n+}. \end{array}$$

X symbolizes the ligand, while M^n+^ represents Cd (II) ions. The concentration of the complex [MX] can be determined using the following equation:

(5) $$\begin{array}{c} {\frac{A}{A_{\text{expt}}}=\frac{\left[\text{MX}\right]}{C}}^{n+}. \end{array}$$

The variable *C* represents the combined concentration of Cd (II) ions and the ligand in the analysis. Therefore, the concentration of the produce complex (MX) can be determined using the equation given below:

(6) $$\begin{array}{c} \left[\text{MX}\right]=\frac{A}{A_{\text{extp}}}.C, \end{array}$$

(7) $$\begin{array}{c} {\left[M\right]}^{n+}=C_{m}-{\left[\text{MX}\right]}^{n+}=C_{m}-\frac{A}{A_{\text{extp}}}.C, \end{array}$$

(8) $$\begin{array}{c} \left[X\right]=C_{x}-{\left[\text{MX}\right]}^{n+}=C_{x}-\frac{A}{A_{\text{extp}}}.C. \end{array}$$

In order to get the created complex's stability constant (K), one can use the following equation:

(9) $$\begin{array}{c} K=\frac{\left[\text{MX}\right]}{\left[M\right].\left[X\right]}. \end{array}$$

The stability constant value was discovered to be (4.33 × 10^2^ mol^−1^ L).

### 3.5. The Conformation and Characterization of the Ligand and L-Cd Complex

#### 3.5.1. Conformation of Ligand With Spectral Data

The ligand (L) was synthesized according to the procedure carried out in our lab and published according to reference [31]. The structure of such ligand was confirmed from their IR and NMR (^1^H and ^13^C) data as follows: One sharp band for NH appeared at 3426 cm^−1^ and two intense sharp absorbtion bands for NH_2_ found at 3251 and 3153 cm^−1^ in IR spectrum. In addition, the carbonyl group of the indole moiety appeared at 1735 cm^−1^ (C = O) and the C = N band located at 1613 and 1601cm^−1^. Moreover, The ^1^H NMR spectrum of the ligand (L) in (DMSO-d_6_ showed three singlet signals for six protons of 2NH and 2NH_2_ groups at *δ = *8.68 (2H), 9.06 (2H), 12.34 (2H) ppm in addition to three multiplet signals at *δ* 2.05 (*p*, 2H) for CH_2_ group, 3.86 (*t*, 4H) for two CH_2_ groups and 7.11–7.68 (*m*, 8H) for aromatic moieties. The ^13^C NMR in CDCl_3_ revealed 11 carbon signals matching with the proved structure of ligand (L) two carbon signal at *δ* = 21.5 and 42 for the two different CH2 groups, six carbon signals from 119 to 142 for the carbons of aromatic of indole moieties, the carbon of C = N appeared at 131, carbon of C = O found at 161 and the characteristics carbon for the C = S group located at 190 ppm.

#### 3.5.2. FTIR Spectrum of Cd (II) Ions-Complex

To confirm the coordination of the CdCl_2_ with the *bis*-thiosemicarbazone ligand, the important absorption peaks in IR spectrum of the Cd (II) ions-complex was studied and compared with the characteristic bands of the ligand's IR spectrum. The IR spectrum of the Cd (II) ions complex showed shift in several absorption bands as *ν* (C = N) appeared at 1620 cm^−1^ that higher than the same band appeared in the ligand at 1601 cm^−1^. This shift suggests that the nitrogen of azomethine group coordinated to Cd (II) ions [24, 34–36]. The thioamide band in the *bis*-thiosemicarbazone ligand shifted from 824 cm^−1^ to 727 cm^−1^ in the Cd (II) ions complex that indicated the connection between C = S through sulfur atom with the metal [36]. Another band appeared at 457 cm^−1^ was referred to *ν* (M-N) frequency [23].

#### 3.5.3. Thermogravimetric Analysis (TGA)

**Figure 9:**
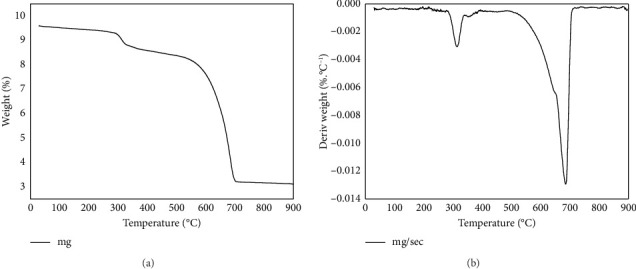
TGA (a) and DTG (b) curves of Cd (II) ions-complex.

TGA is a reliable technique for assessing the heat stability of metal chelates. It provides typical information about these metal complexes' thermal properties and decomposition methods, allowing for a better understanding of their chemical composition. The Cd (II) ions-complex was subjected to TGA analyses and the results illustrated in Figure 9. The examination the thermogravimetry of the Cd (II) ions-complex showed that this complex underwent three steps for thermal decomposition in the range 32°C–901°C. The degradation process of Cd (II) ions complex illustrated as behind:

(10) $$\begin{array}{c} \begin{array}{c} \left[L{\text{Cd}}_{2}{\text{Cl}}_{4}{\left(H_{2}O\right)}_{2}\right]\\ \text{Mwt}=883.23 \end{array}\overset{32-298{}^{\circ}C}{\overset{⟶}{-3.65\%\;\left(\text{calcd}.\;4.07\%\right)}}\begin{array}{c} \left[L{\text{Cd}}_{2}{\text{Cl}}_{4}\right]\\ \text{Mwt}=847.20 \end{array},\\ \begin{array}{c} \left[L{\text{Cd}}_{2}{\text{Cl}}_{4}\right]\\ \text{Mwt}=847.20 \end{array}\overset{299-544{}^{\circ}C}{\overset{⟶}{-10.52\;\left(\text{calcd}.\;10.15\%\right)}}\begin{array}{c} \left[L{\text{Cd}}_{2}{\text{Cl}}_{2}-{\text{NH}}_{2}\right]\\ \text{Mwt}=761 \end{array},\\ \begin{array}{c} \left[L{\text{Cd}}_{2}{\text{Cl}}_{2}-{\text{NH}}_{2}\right]\\ \text{Mwt}=761 \end{array}\overset{544-901{}^{\circ}C}{\overset{⟶}{-53.17\;\left(\text{calcd}.\;52.56\%\right)}}\begin{array}{c} 2\left(\text{CdS}\right)+6C\\ \text{Mwt}=361 \end{array}. \end{array}$$

The first step of the decomposition appeared at 32°C–298°C assigned to loss of the coordinated two water molecules. Through the second step from 299°C–544°C refereed to the separation of two Cl atoms with NH_2_ group. The last third stage appeared at 544°C–901°C assigned to the degradation of the organic ligands with the last two Cl atoms leading to the formation of two Cd (II) ions with carbon residues. The data of IR and thermal analysis confirm the Cd (II) ions Complex has the molecular formula (LCd_2_Cl_4_ [H_2_O]_2_).

## 4. Conclusion

For the goal of spectrophotometric detection of Cd (II) ions, a new ligand has been discovered. The process hinged on using the reagent to produce a long-lasting yellow-orange compound at a pH of 12. The 290 nm wavelength was the one that revealed the complex absorption. With a correlation coefficient of 0.9869, the concentration range of (1.8–17.8 mol L^−1^) showed linearity. There was a LOQ of 0.815 μg mL^−1^ (7.3 μmol L^−1^) and a LOD of 0.245 μg mL^−1^ (7.3 μmol L^−1^). The correlation between the findings obtained from Job's continuous variation approach and his molar-ratio method shows that the complex has a stoichiometric composition of 2:1 of M:L. In addition, we have recovered the solid complex that forms when L reacts with CdCl_2_, and we have used alternative ways to study its structure. The results show that the Cd (II) ions complex has formed with the same molecular ratio as in the solution, and the formula is (LCd_2_Cl_4_ [H_2_O]_2_).

## Figures and Tables

**Table 1:** Quantitative parameters for determination of Cd (II) ions complex.

Parameters	Values
pH	12
*λ*_max_	290 nm
Molar ratio (ligand-Cd (II) ions)	2:1
Beers law limits (× 10^−5^ mol L^−1^)	1.8 – 17.8
Molar absorptivity (L mol^−1^ cm^−1^)	6.7 × 10^2^
Regression equation	*A* = 0.0067C + 0.1268
Intercept	0.1268
Slope	0.0067
Correlation coefficient (*r*^2^)	0.9869
RSD %	5.1%
LOD (μg mL^−1^)	0.245
LOQ (μg mL^−1^)	0.817

## Data Availability

Data will be made available on request.
